# Supplementary material for: Sphingosine simultaneously inhibits nuclear import and activates PP2A by binding importins and PPP2R1A
Source: EMBO J. 2025 Jun 30;44(16):4473–98. doi: 10.1038/s44318-025-00490-5 (PMC12361511; doi:10.1038/s44318-025-00490-5)
Supplement: Supplementary file 1 — Appendix [file 44318_2025_490_MOESM1_ESM.pdf]

APPENDIX for

Sphingosine simultaneously inhibits nuclear import and activates PP2A by binding importins and PPP2R1A

Table of contents

Appendix Figure S1 1
Appendix Figure S2 3
Appendix Figure S3 4
Appendix Figure S4 6
Appendix Figure S5 8
Appendix Figure S6 9
Appendix Figure S7 10
Appendix Figure S8 12
Appendix Figure S9 14
Appendix Figure S10 16

Table with exact P values for all figures 18

Appendix Figure S1

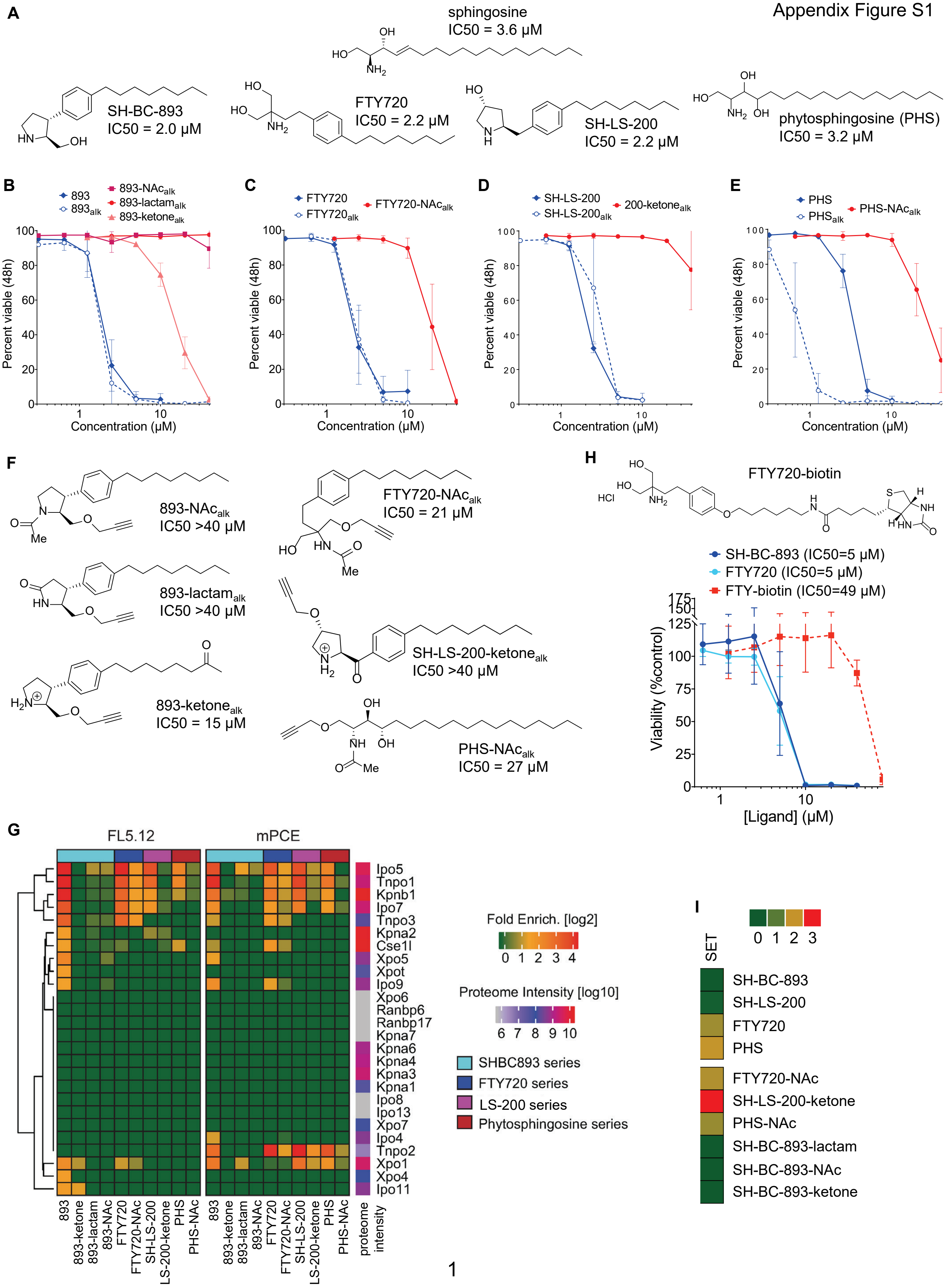

Appendix Figure S1: Unbiased chemoproteomics using 4 active and 6 inactive sphingosine analogs identifies PPP2R1A and importins, but not SET, as targets.

A Structures of sphingosine and the unmodified active ligands.

B-E IC50 curves for unmodified active ligands and alkyne-modified active and inactive compounds in FL5.12 cells at 48 h; mean ± SD, n = 3-5.

F Inactive compounds and their IC50 in FL5.12 cells at 48 h; n ≥ 3.

G Protein enrichment in chemoproteomics for each cell type shown by compound and by karyopherin; mean is shown, n=3.

H IC50 curves in mPCE prostate cancer cells at 72 h for SH-BC-893, FTY720, or FTY720-biotin; mean ± SD, n=3-4.

I SET protein enrichment in chemoproteomics using FL5.12 lysates shown for each of the ligands relative to unconjugated beads; mean is shown, n=3.

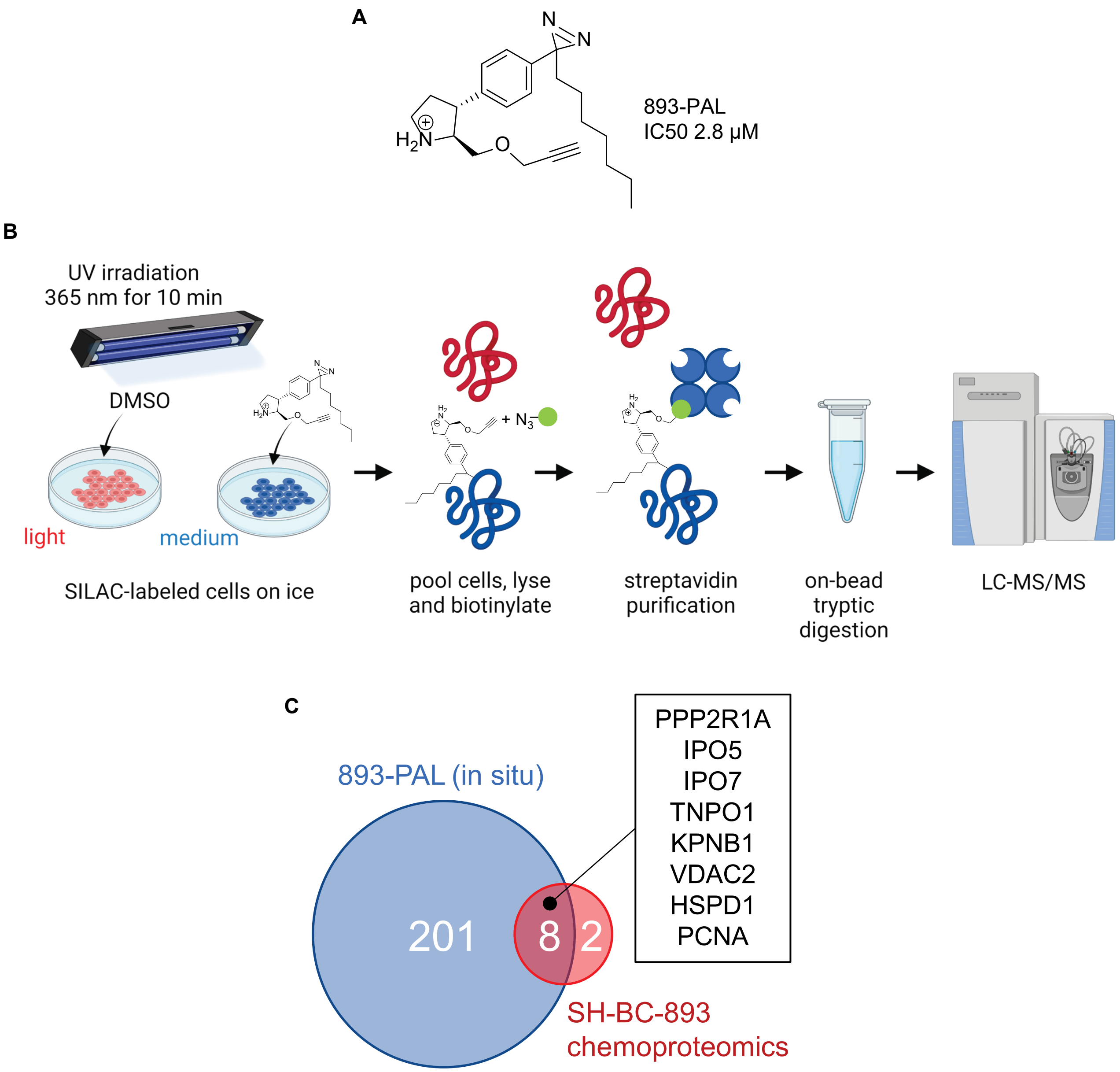

Appendix Figure S2: A photoaffinity labeling (PAL) probe confirms that SH-BC-893 binds PPP2R1A and importins in intact cells.

A Structure of 893-PAL probe and its IC50 in FL5.12 cells at 48 h (mean, n = 3).

B Scheme for PAL experiments.

C 203 (FL5.12) or 60 (mPCE) proteins were significantly enriched by the 893-PAL probe (P value < 0.01) representing 209 different proteins. Eight of the proteins enriched by 893-PAL were also enriched >8-fold by SH-BC-893 relative to its inactive analogs in chemoproteomics assays while 2 proteins were isolated by chemoproteomics but not PAL. See also Dataset EV2.

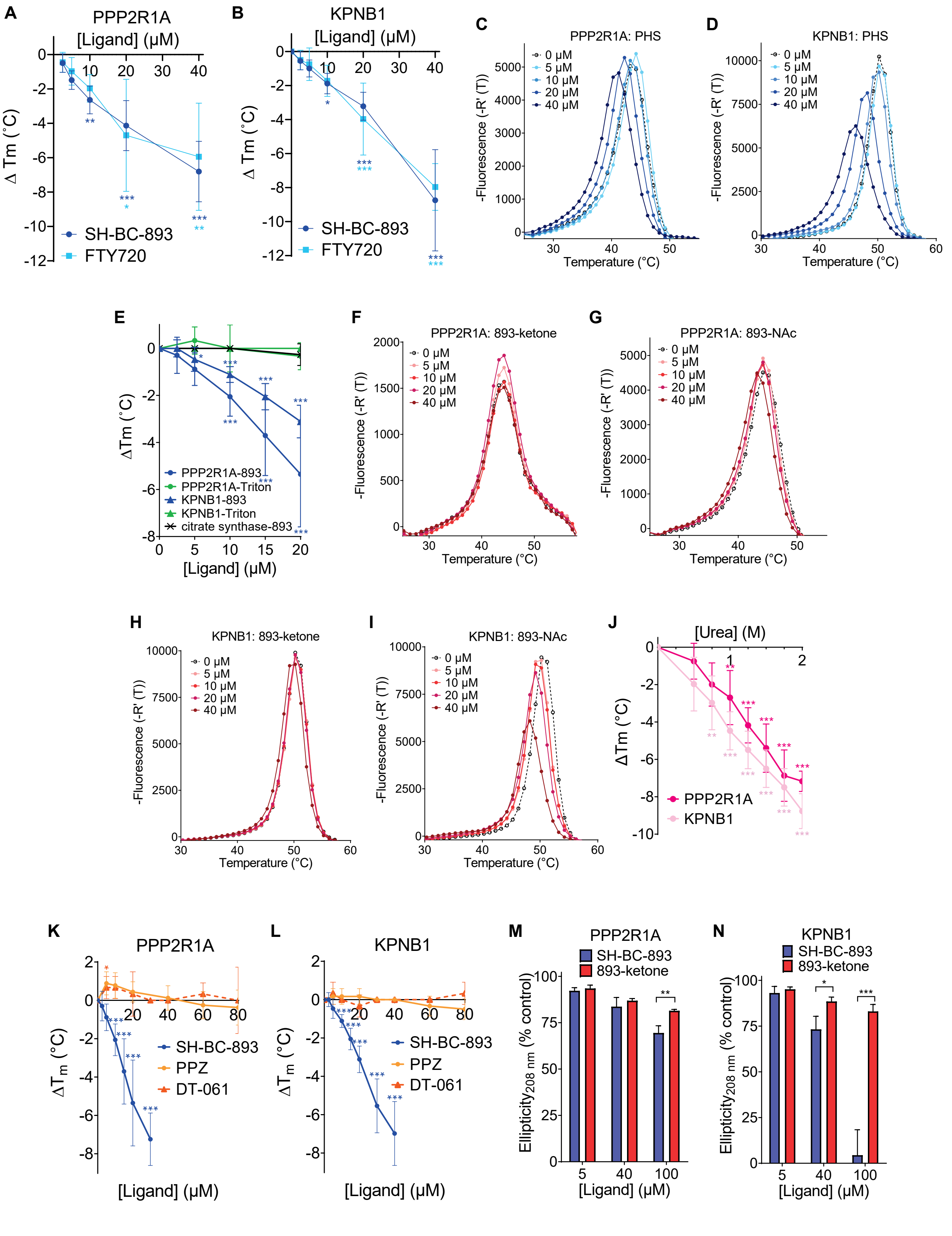
A
PPP2R1A
[Ligand] (μM)
Δ Tm (°C)
SH-BC-893
FTY720
B
KPNB1
[Ligand] (μM)
Δ Tm (°C)
SH-BC-893
FTY720
C
PPP2R1A: PHS
0 μM
5 μM
10 μM
20 μM
40 μM
-Fluorescence (-R' (T))
Temperature (°C)
D
KPNB1: PHS
0 μM
5 μM
10 μM
20 μM
40 μM
-Fluorescence (-R' (T))
Temperature (°C)
E
ΔTm (°C)
PPP2R1A-893
PPP2R1A-Triton
KPNB1-893
KPNB1-Triton
citrate synthase-893
[Ligand] (μM)
F
PPP2R1A: 893-ketone
0 μM
5 μM
10 μM
20 μM
40 μM
-Fluorescence (-R' (T))
Temperature (°C)
G
PPP2R1A: 893-NAc
0 μM
5 μM
10 μM
20 μM
40 μM
-Fluorescence (-R' (T))
Temperature (°C)
H
KPNB1: 893-ketone
0 μM
5 μM
10 μM
20 μM
40 μM
-Fluorescence (-R' (T))
Temperature (°C)
I
KPNB1: 893-NAc
0 μM
5 μM
10 μM
20 μM
40 μM
-Fluorescence (-R' (T))
Temperature (°C)
J
[Urea] (M)
ΔTm (°C)
PPP2R1A
KPNB1
K
PPP2R1A
ΔTm (°C)
SH-BC-893
PPZ
DT-061
[Ligand] (μM)
L
KPNB1
ΔTm (°C)
SH-BC-893
PPZ
DT-061
[Ligand] (μM)
M
PPP2R1A
SH-BC-893
893-ketone
Ellipticity208 nm (% control)
[Ligand] (μM)
N
KPNB1
SH-BC-893
893-ketone
Ellipticity208 nm (% control)
[Ligand] (μM)

Appendix Figure S3: Active, but not inactive, sphingosine-like compounds bind to PPP2R1A and KPNB1 inducing conformational change.

A-D Thermal shift assays using recombinant PPP2R1A or KPNB1.1 and the indicated concentrations of SH-BC-893, FTY720, or phytosphingosine (PHS). Mean ± SD shown for A,B and representative first derivative plots for C,D; n=3-9 biological replicates (each biological replicate is a mean of 4 technical replicates).

E Effect of SH-BC-893 (blue) or Triton X-100 (Triton, green) on the T_m of PPP2R1A, KPNB1.1, or citrate synthase in thermal shift assays. Mean ± SD shown; n=3-17.

F-I As in (A-D) but with inactive ligands. Representative first derivative plots shown; n=2-7.

J Effect of increasing concentrations of urea on the T_m of PPP2R1A or KPNB1.1 in thermal shift assays. Mean ± SD shown; n=4.

K,L Effect of the PP2A agonists perphenazine (PPZ) or DT-061 (also known as SMAP) on the T_m of PPP2R1A (K) or KPNB1.1 (L), SH-BC-893 shown for comparison. Mean ± SD shown; n=3-17.

M,N Ellipticity at 208 nm in circular dichroism assays with recombinant PPP2R1A (M) or KPNB1.1 (N). Mean ± SD is shown; n=3-6.

Comparing treated samples to the untreated control using a one-way ANOVA with Dunnett's correction (A,B,E, and J-L) or comparing results with SH-BC-893 and the inactive control 893-ketone using multiple unpaired t tests (M and N), ***, P ≤ 0.001; **, P ≤ 0.01; *, P ≤ 0.05. See appendix table for exact P values.

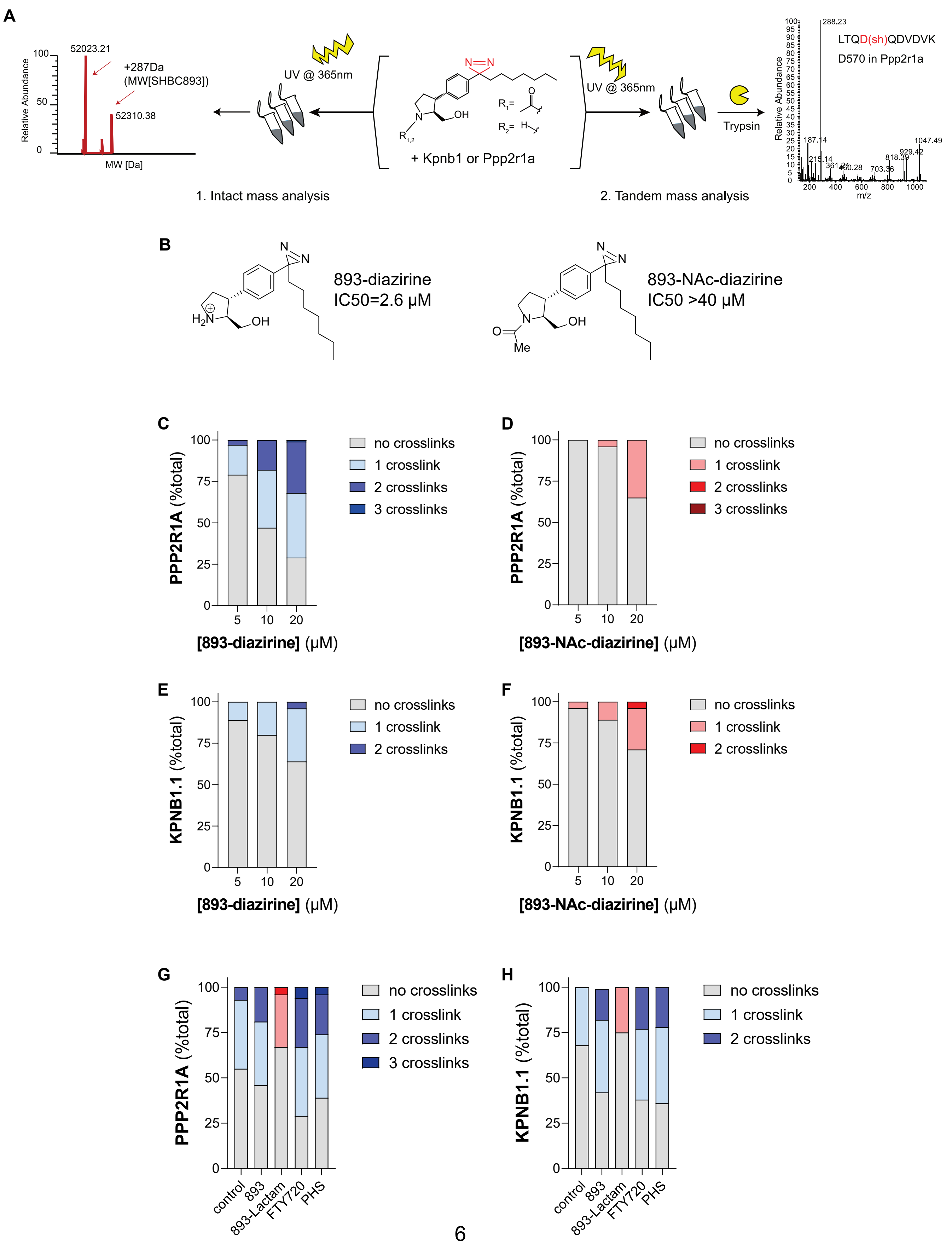
A
Relative Abundance
MW [Da]
52023.21
+287Da
(MW[SHBC893])
52310.38
UV @ 365nm
R1=
R2= H
OH
+ Kpnb1 or Ppp2r1a
UV @ 365nm
Trypsin
LTQD(sh)QDVDVK
D570 in Ppp2r1a
288.23
187.14
215.14
1047.49
929.42
818.39
703.36
m/z
1. Intact mass analysis
2. Tandem mass analysis
B
893-diazirine
IC50=2.6 µM
893-NAc-diazirine
IC50 >40 µM
C
PPP2R1A (%total)
[893-diazirine] (µM)
no crosslinks
1 crosslink
2 crosslinks
3 crosslinks
D
PPP2R1A (%total)
[893-NAc-diazirine] (µM)
no crosslinks
1 crosslink
2 crosslinks
3 crosslinks
E
KPNB1.1 (%total)
[893-diazirine] (µM)
no crosslinks
1 crosslink
2 crosslinks
F
KPNB1.1 (%total)
[893-NAc-diazirine] (µM)
no crosslinks
1 crosslink
2 crosslinks
G
PPP2R1A (%total)
control
893
893-Lactam
FTY720
PHS
no crosslinks
1 crosslink
2 crosslinks
3 crosslinks
H
KPNB1.1 (%total)
control
893
893-Lactam
FTY720
PHS
no crosslinks
1 crosslink
2 crosslinks

Appendix Figure S4: Intact mass measurements of SH-BC-893 crosslinking to PPP2R1A or KPNB1.

A Schematic showing mass spectrometry methods used to monitor crosslinking of 893-diazirine and 893-NAc-diazirine to PPP2R1A and KPNB1.1.

B Structures of 893-diazirine or 893-NAc-diazirine with their IC50 in FL5.12 cells at 48 h. Mean shown, n=3.

C-F Results of intact mass analysis after UV-crosslinking the indicated amount of 893-diazirine (C,E) or 893-NAc-diazirine (D,F) to 2 µM PPP2R1A (C,D) or KPNB1.1 (E,F). Crosslinks indicate the number of diazirine analog molecules covalently linked to the protein; conjugation can take place at multiple sites. See also Datasets EV3 and EV4.

G,H As in (C,E), crosslinking was performed with 10 µM 893-diazirine but in the presence of DMSO (control) or 10 µM of the indicated unmodified compounds. See also Dataset EV5. Active compounds depicted in blue and inactive compounds in red as in main text.

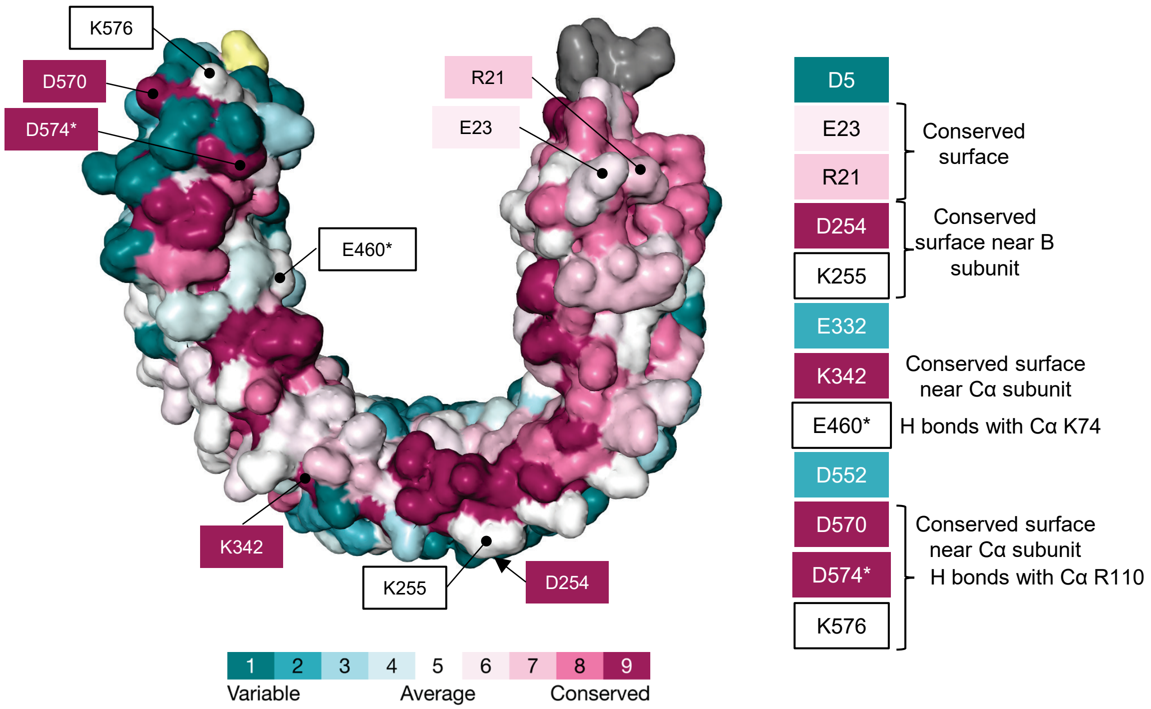

Appendix Figure S5: 893-diazirine crosslinks to conserved regions in PPP2R1A. Tandem mass spectrometry was used to identify the amino acids in recombinant PPP2R1A that crosslinked to 5 μM 893-diazirine. Conservation determined using ConSurf with default settings, asterisks indicate amino acids that are involved in inter-subunit hydrogen bonding. See also Dataset EV6. Crosslinking sites are shown on PDB 2IAE, a crystal structure of the complex of PPP2R1A (colored to show conservation), PPP2CA (Cα, not shown), PPP2R5C (B56γ, not shown), and microcystin (not shown). PPP2R1A is shown rotated so the main inter-subunit interface is facing the viewer.

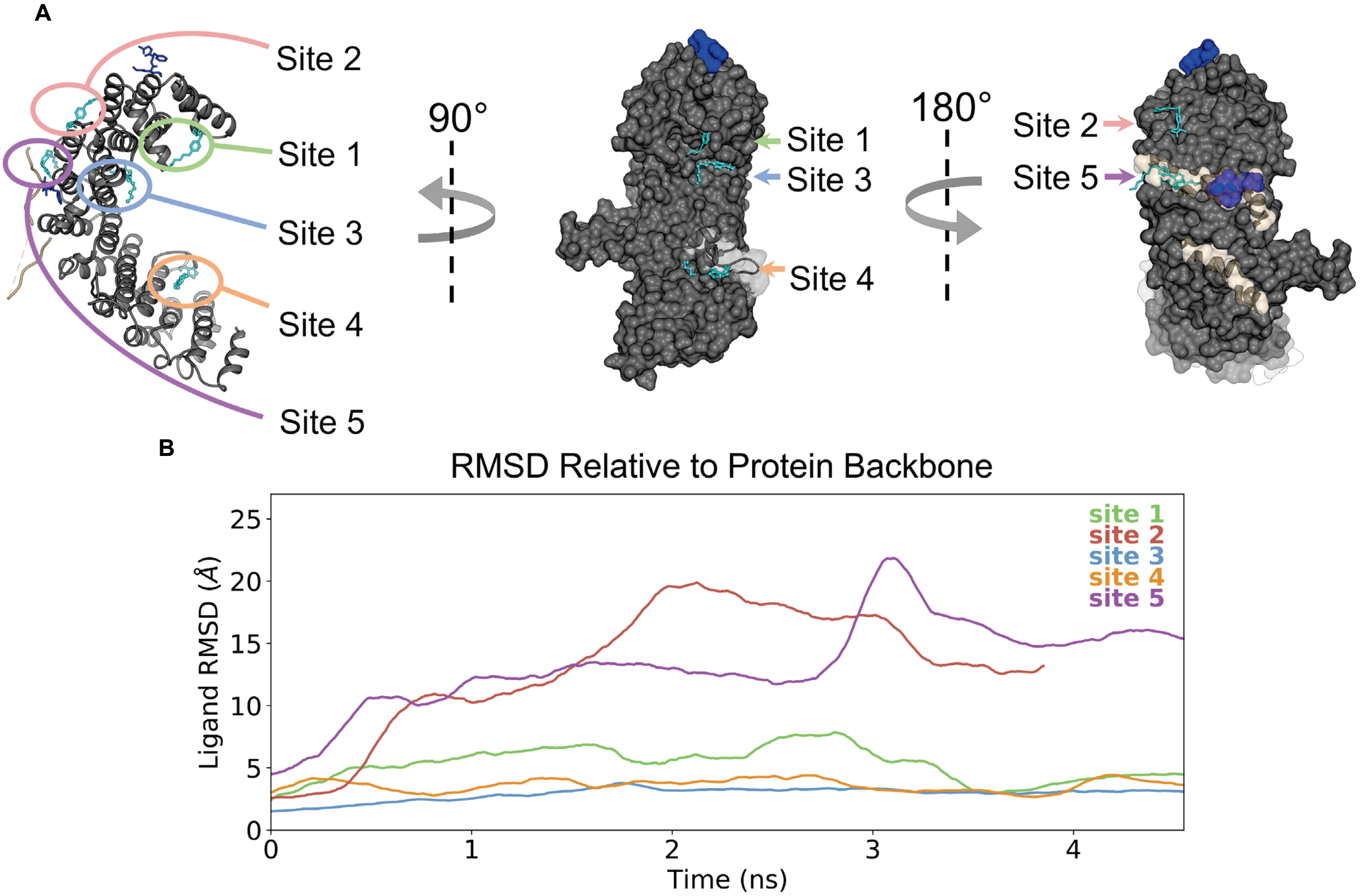

Appendix Figure S6: Molecular dynamics simulations suggest that SH-BC-893 may disrupt KPNB1 association with KPNA2.

A The starting configurations of SH-BC-893 bound to KPNB1 from PBD ID 3LWW for molecular dynamics simulations. The simulated sites are labeled 1 through 5. KPNB1 is shown in dark grey, and the binding regions of fragments of GLFG NPC in blue (excluded from simulation). Sites 1, 3, and 4 are on the inner curve of KPNB1.

B The heavy ligand atom root-mean-square deviation, RMSD, over simulation time with respect to the protein backbone. Sites 3 and 4 show sustained ligand binding through the simulation.

A

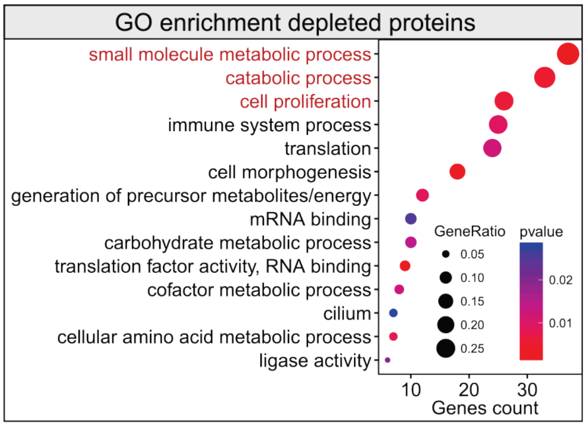

B

Oncogenic cargo of sphingosine-binding importins

	KPNB1	TNPO1	IPO7	IPO5
MYC	+			
AR	+			
hnRNPA1		+		
YAP			+	
JUN	+	+	+	+
ribosomal proteins	+	+	+	+
NF-κB	+			

C

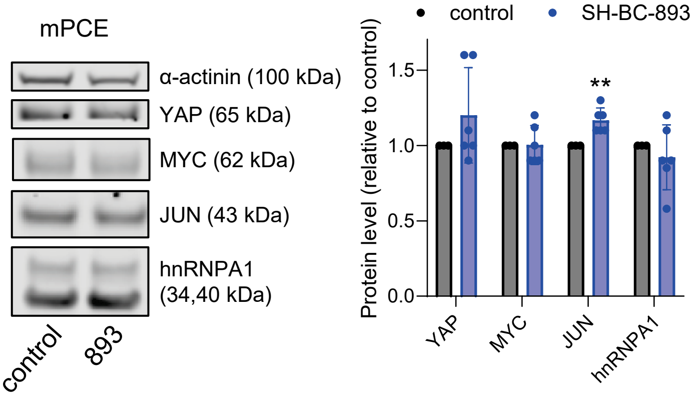

D

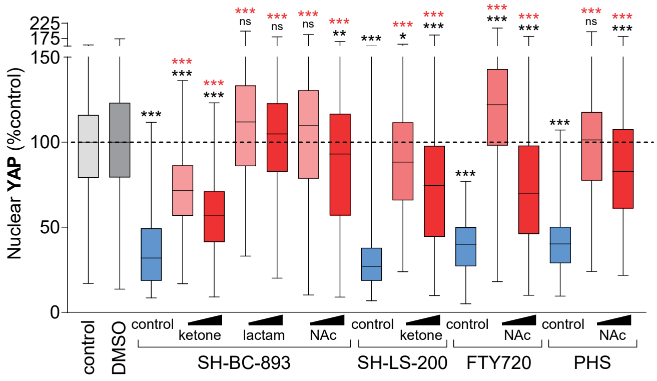

E

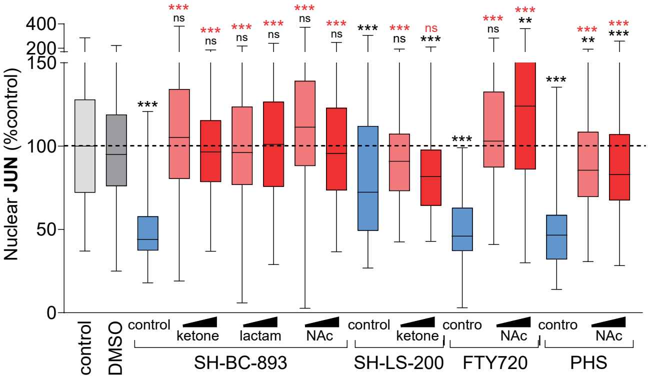

Appendix Figure S7: Sphingosine-like compounds reduce the nuclear levels of proteins that promote tumor growth and progression.

A Gene ontology analysis of proteins lost from the nucleus of SH-BC-893-treated mPCE cells.

B Table summarizing cargos discussed in this manuscript and transported by the affected importins.

C Protein levels measured by western blot in mPCE treated with 5 µM SH-BC-893 for 6 h. Bands were quantified, expressed relative to the loading control α-actinin, and then normalized to control. Mean ± SD shown; n=3-6. P>0.05 except for JUN where P=0.01 using an unpaired t test to compare ± 893. Representative blot shown.

D,E Nuclear levels of YAP (D) or JUN (E) in mPCE cells 6 h after treatment with the indicated active (blue) or inactive (red) analogs; 171-327 cells from one biological replicate evaluated. Median and quartiles shown. The D'Agostino and Pearson test was used to evaluate whether data was normally distributed. Box-and-whisker plots display the median and interquartile range (box), as well as the range of the lower and upper quartiles (whiskers). Using a Kruskal-Wallis test with Dunn's multiple comparison test, ***, P ≤ 0.001; **, P ≤ 0.01; *, P ≤ 0.05; ns, not significant, P > 0.05. See appendix table for exact P values. Black asterisks compare results to the vehicle control and red asterisks compare results to the active congener.

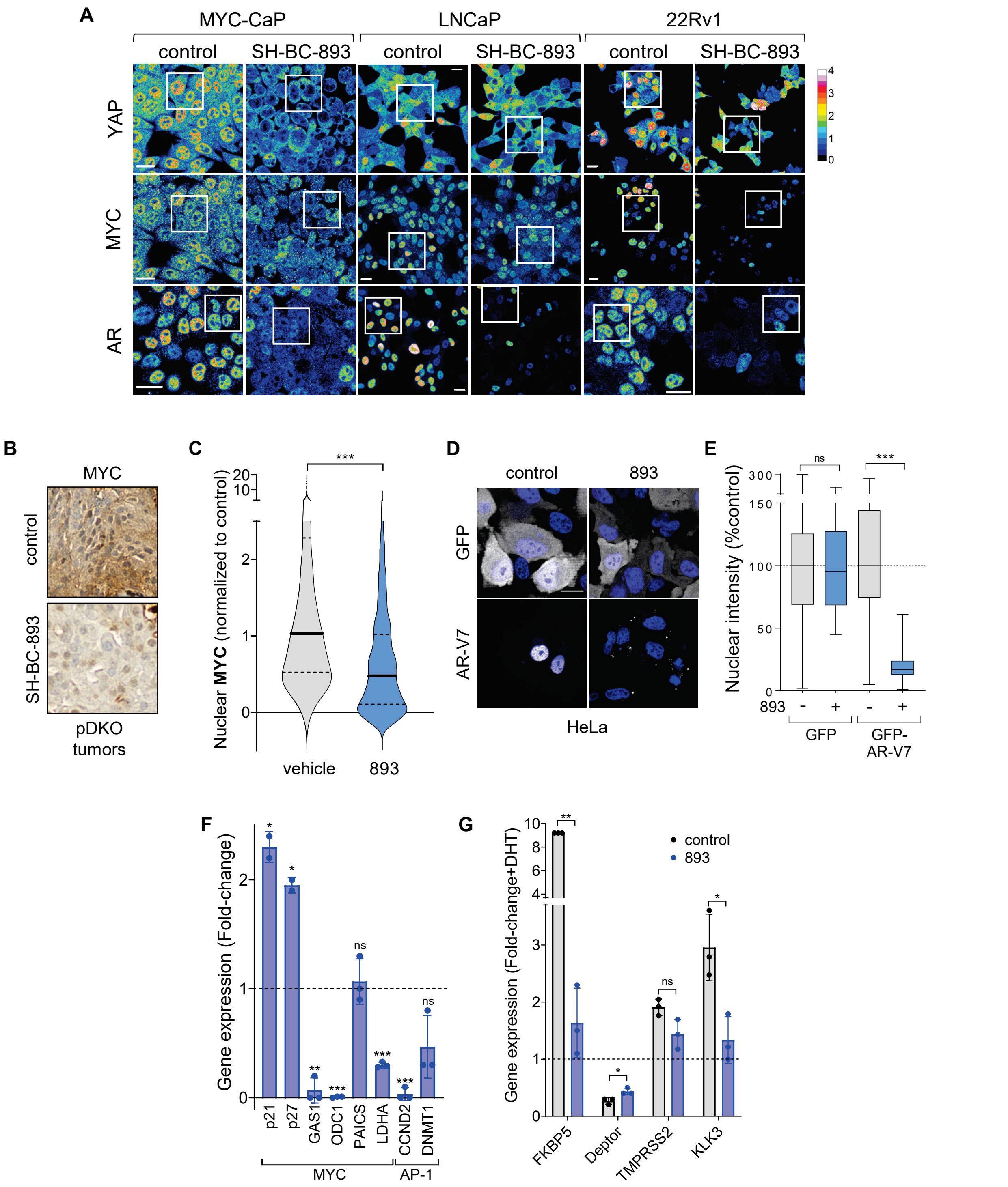
A
MYC-CaP
LNCaP
22Rv1
control
SH-BC-893
YAP
MYC
AR
B
MYC
control
SH-BC-893
pDKO tumors
C
***
Nuclear MYC (normalized to control)
vehicle
893
D
control
893
GFP
AR-V7
HeLa
E
ns
***
Nuclear intensity (%control)
893
GFP
GFP-AR-V7
F
Gene expression (Fold-change)
p21
p27
GAS1
ODC1
PAICS
LDHA
CCND2
DNMT1
MYC
AP-1
G
Gene expression (Fold-change+DHT)
control
893
FKBP5
Deptor
TMPRSS2
KLK3

Appendix Figure S8: Sphingosine-like compounds reduce the nuclear levels and activity of proteins that promote tumor growth and progression.

A Larger field of view for images shown in main Figure 3D.

B,C Nuclear MYC levels as detected by IHC in tumors from *PB4-Cre;p53$^{flox/flox}$;Pten$^{flox/flox}$* (pDKO) mice treated with vehicle (water) or 120 mg/kg SH-BC-893 QD. In (C), tumors in 3 different mice were evaluated, a total of >2,700 (control) or 3,102 (893) nuclei quantified; median (solid line) and quartiles (dashed lines) shown. Background (average from a sample not exposed to primary antibody) subtracted from all samples. Using D'Agostino & Pearson test, the datasets were found not to be normally distributed (P<0.0001) and thus an upaired Mann-Whitney test was employed, P <0.0001.

D,E Nuclear GFP levels in HeLa cells stably expressing GFP or GFP-AR-V7 ± SH-BC-893 (5 μM) for 6 h. In (E), box-and-whisker plots display the median and interquartile range (box), as well as the range of the lower and upper quartiles (whiskers); 50 cells from 2 biological replicates evaluated. Using D'Agostino & Pearson test, the datasets were found not to be normally distributed (P<0.0001) and thus an upaired Mann-Whitney test was employed.

F MYC and AP-1 target gene (mRNA) expression in 22Rv1 cells maintained in complete medium lacking DHT, fold-change compares vehicle and SH-BC-893 (10 μM) at 24 h. p21 and p27 are generally suppressed by MYC, other genes are positively regulated by MYC or AP-1. Mean ± SD from 3 biological replicates shown except for p21/27 where an outlier with a large positive fold change (~8) was discarded thus mean and range shown, n=2.

G After a 12 h serum withdrawal, 22Rv1 cells were treated with SH-BC-893 (10 μM) for 6 h before DHT (10 nM) or vehicle (ethanol) was added for an additional 16 h. The levels of the indicated mRNA were determined in 3 biological replicates using RT-qPCR; means ± SD shown.

Using multiple unpaired Welch's t tests due to unequal SDs in F and G, ***, P ≤ 0.001; **, P ≤ 0.01; *, P ≤ 0.05; ns, not significant, P > 0.05. See Appendix table for exact P values. Scale bars, 20 μm.

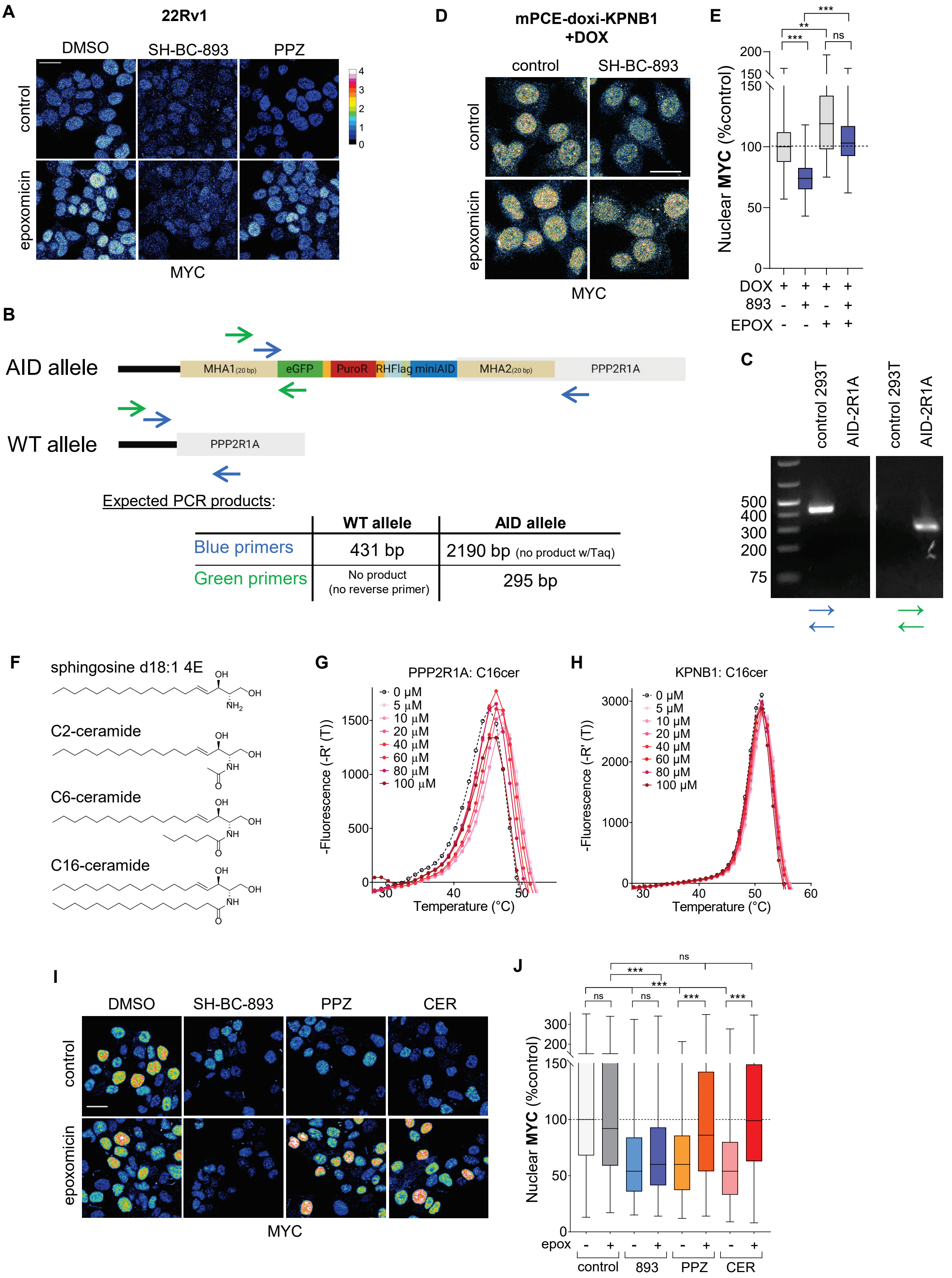
A
22Rv1
DMSO
SH-BC-893
PPZ
control
epoxomicin
MYC
D
mPCE-doxi-KPNB1
+DOX
control
SH-BC-893
control
epoxomicin
MYC
E
Nuclear MYC (%control)
DOX + + + +
893 - + - +
EPOX - - + +
B
AID allele
MHA1(20 bp)
eGFP
PuroR
RHFlag
miniAID
MHA2(20 bp)
PPP2R1A
WT allele
PPP2R1A
Expected PCR products:
WT allele
AID allele
Blue primers
431 bp
2190 bp (no product w/Taq)
Green primers
No product (no reverse primer)
295 bp
C
control 293T
AID-2R1A
control 293T
AID-2R1A
500
400
300
200
75
F
sphingosine d18:1 4E
C2-ceramide
C6-ceramide
C16-ceramide
G
PPP2R1A: C16cer
-Fluorescence (-R' (T))
Temperature (°C)
0 µM
5 µM
10 µM
20 µM
40 µM
60 µM
80 µM
100 µM
H
KPNB1: C16cer
-Fluorescence (-R' (T))
Temperature (°C)
I
DMSO
SH-BC-893
PPZ
CER
control
epoxomicin
MYC
J
Nuclear MYC (%control)
epox - + - + - + - +
control
893
PPZ
CER

Appendix Figure S9: The parallel actions of SH-BC-893 on PPP2R1A and KPNB1 provide resistance to disruption of PP2A-dependent degradation.

A Endogenous MYC localization in 22Rv1 cells treated with DMSO, 10 µM SH-BC-893, or 25 µM perphenazine (PPZ) ± 100 nM epoxomicin for 6 h.

B Creation of 293T-AID-2R1A cells. Wild type (WT) and knock-in alleles shown. MHA1/2, major homology arm 1 or 2; eGFP, enhanced green fluorescent protein; T2A and P2A, 2A peptides that induces ribosomal skipping during translation; PuroR, puromycin resistance gene; FLAG, FLAG epitope tag; miniAID, miniature auxin inducible degron domain; SV40, SV40 polyoma virus promoter; H, hygromycin resistance gene. The PPP2R1A protein produced from this allele is N-terminally tagged with FLAG and the AID domain. Two different primer pairs designed to detect the wildtype (blue) or knock-in (green) alleles are shown.

C PCR performed on genomic DNA confirming homozygous knock-in of AID-2R1A construct. PCR using the blue primers produces a 431 bp (wild type allele) or 2190 bp product (modified allele, too big to amplify under these PCR conditions). PCR with the green primers produces a 295 bp product only from the knock-in allele. Left lane, markers with 500 bp band labeled, other bands are 1,000, 700, 400, 300, 200, or 75 bp.

D,E mPCE-doxi-KPNB1 cells were treated overnight with doxycycline and then for 6 h with SH-BC-893 (5 µM) and/or epoxomicin (100 nM) as indicated. In (D), intensity scale as in (A). In (E), median and quartiles shown; >60 cells from one biological replicate examined.

F-H structures of sphingosine and ceramides. C16 ceramide was evaluated in thermal shift assays with recombinant PPP2R1A (G) or KPNB1.1 (H); n=2, representative curves are shown and the indicated.

I,J 22Rv1 cells were treated for 6 h with epoxomicin (100 nM), SH-BC-893 (10 µM), perphenazine (25 µM), and/or C2-ceramide (50 µM) as indicated and nuclear MYC quantified. In (I), intensity scale as in (A). In (J), box-and-whisker plots display the median and interquartile range (box), as well as the range of the lower and upper quartiles (whiskers); 355-415 cells from two biological replicates examined.

Using a D'Agostino and Pearson normality test followed by a Kruskal-Wallis test with Dunn's multiple comparisons test (C,J), ***, $P \leq 0.001$; **, $P \leq 0.01$; ns, not significant $P > 0.05$. See appendix table for exact P values. Scale bars, 20 µm.

A

Formula for calculating basal sphingosine concentration in WT MEFs:

$$\frac{50\ fmol}{\mu g\ protein} \times \frac{1\ \mu g\ protein}{1{,}000{,}000\ pg\ protein} \times \frac{590\ pg\ protein}{2{,}263\ fL} = 13\ x\ 10^{-6}\ M\ (\ or\ \frac{fmol}{fL})\ or\ 13\ \mu M$$

CELL TYPE	[SO] fmol/μg protein	pg protein per cell	mean cell volume (fl)	[SO] μM
SK1 WT MEF	50	590	2,263	13
SK1 KO MEF	57	560	2,155	15
HeLa	113	333	2,327	16
ACER1 HeLa	18	357	1,672	4
22Rv1	12.2	440	1,205	4

B

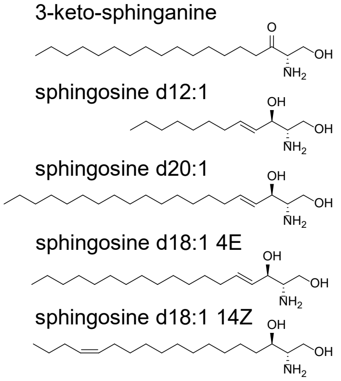

C

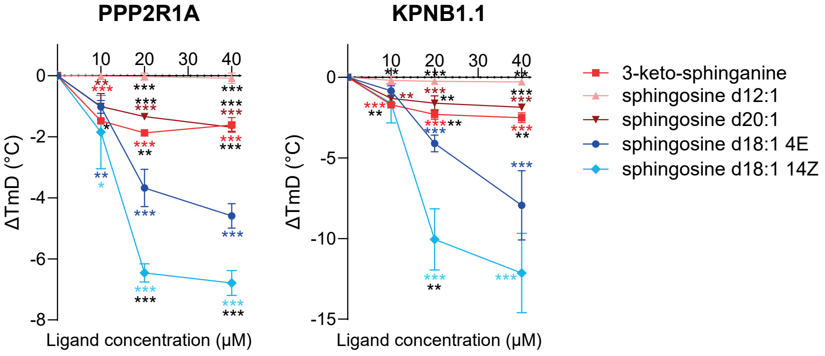

D

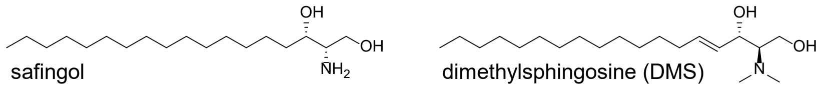

E **F** **G** **H**

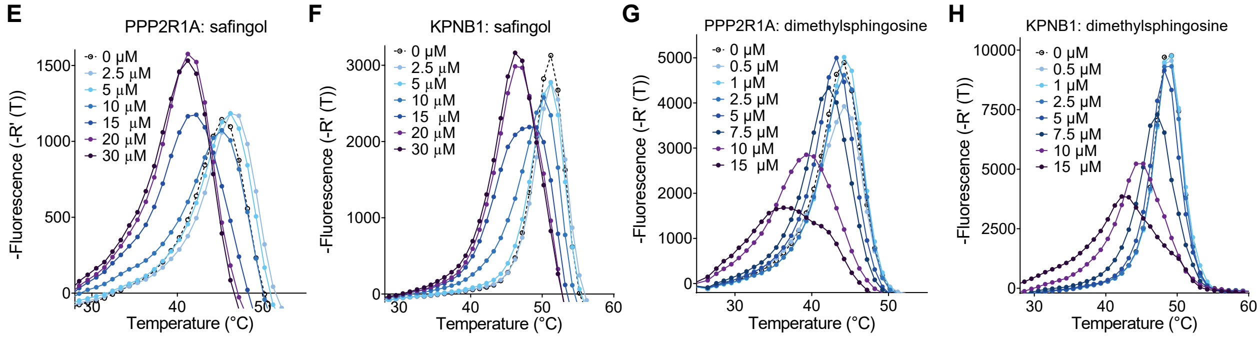

I

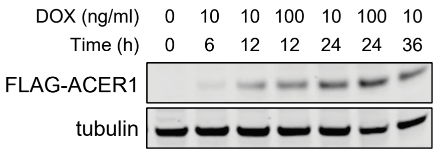

J

FLAG-ACER1 HeLa

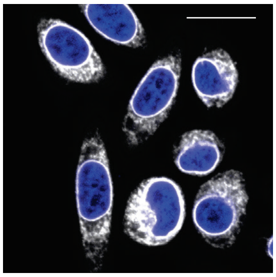

Appendix Figure S10: Sphingosine-like SK1 inhibitors but not all sphingosine variants engage the same targets as SH-BC-893.

A Method used to calculate sphingosine molarity in the cells in this study. Sphingosine (fmol/µg protein) measured by LC-MS/MS, protein per cell by BCA assay, and cell volume by Coulter counter.

B,C Structures of sphingosine and related molecules (B). Thermal shift assays (C) using recombinant PPP2R1A or KPNB1.1 with the indicated compounds. Mean ± SD shown, n=3-4 biological replicates (each a mean of 4 technical replicates). Multiple unpaired t tests were performed; ***, $P \leq 0.001$; **, $P \leq 0.01$; *, $P \leq 0.05$, non-significant comparisons ($P>0.05$) are not marked. See appendix table for exact P values. Colored asterisks denote tests relative to the DMSO control for that compound, while black asterisks represent comparisons to the endogenous sphingosine (d18:1 4E) control at the same concentration.

D-H Structures (D) of sphingosine analogs used in the literature as SK inhibitors. Thermal shift assays using recombinant PPP2R1A (E,G) or KPNB1.1 (F,H) and the indicated concentrations safingol or DMS. Representative first derivative plots shown; n=4.

I Western blot showing the induction of FLAG-ACER1 by doxycycline over time. Representative blot shown, n=3.

J Anti-FLAG staining of ACER1-TET-ON HeLa cells 24 h after treatment with 10 ng/ml doxycycline; ACER1 is FLAG-tagged. Scale bar, 20 µm.

EXACT P VALUES FOR ALL FIGURES

	Adjusted P value	significance
Figure 1F		
0 μM vs. 10 μM 893	<0.0001	****
0 μM vs. 15 μM 893	<0.0001	****
0 μM vs. 20 μM 893	<0.0001	****
0 μM vs. 20 μM 893-NAc	0.0005	***
Figure 1H		
0 μM vs. 5 μM 893	0.0189	*
0 μM vs. 10 μM 893	<0.0001	****
0 μM vs. 15 μM 893	<0.0001	****
0 μM vs. 20 μM 893	<0.0001	****
0 μM vs. 20 μM 893-NAc	0.0013	**
Figure 3C		
**YAP:**		
control vs. 893	<0.0001	***
control vs. perphenazine	<0.0001	***
893 vs. perphenazine	<0.0001	***
**JUN:**		
control vs. 893	<0.0001	***
control vs. perphenazine	0.9999	ns
893 vs. perphenazine	<0.0001	***
**hnRNPA1:**		
control vs. 893	<0.0001	***
control vs. perphenazine	0.0007	***
893 vs. perphenazine	<0.0001	***
**MYC:**		
control vs. 893	<0.0001	***
control vs. perphenazine	<0.0001	***
893 vs. perphenazine	0.9999	ns
Figure 3E - YAP		
MYC-CaP control vs. 893	<0.0001	***
LNCaP control vs. 893	<0.0001	***
22Rv1 control vs. 893	<0.0001	***
Figure 3F - MYC		
MYC-CaP control vs. 893	<0.0001	***
LNCaP control vs. 893	<0.0001	***
22Rv1 control vs. 893	<0.0001	***
Figure 3G - AR		
MYC-CaP control vs. 893	<0.0001	***
LNCaP control vs. 893	<0.0001	***
22Rv1 control vs. 893	<0.0001	***
Figure 4C - AR		
control vs. epoxomicin	0.0030	**
control vs. 893	<0.0001	***
control vs. perphenazine	<0.0001	***
893 vs. 893 epoxomicin	>0.9999	ns
perphenazine vs. perphenazine epoxomicin	<0.0001	***
epoxomicin vs. 893 epoxomicin	<0.0001	***
epoxomicin vs. perphenazine epoxomicin	0.0127	*
893 vs. perphenazine	>0.9999	ns
893 epoxomicin vs. perphenazine epoxomicin	<0.0001	***
Figure 4D - MYC		
control vs. epoxomicin	<0.0001	***
control vs. 893	<0.0001	***
control vs. perphenazine	<0.0001	***
893 vs. 893 epoxomicin	0.1058	ns

perphenazine vs. perphenazine epoxomicin<br>epoxomicin vs. 893 epoxomicin<br>epoxomicin vs. perphenazine epoxomicin<br>893 vs. perphenazine<br>893 epoxomicin vs. perphenazine epoxomicin	<0.0001<br><0.0001<br><0.0001<br>>0.9999<br><0.0001	***<br>***<br>***<br>ns<br>***
Figure 4G		
control vs. NAA<br>control vs. 893<br>control vs. perphenazine<br>893 vs. 893 NAA<br>893 vs. perphenazine<br>perphenazine vs. perphenazine NAA<br>control NAA vs. 893 NAA<br>control NAA vs. perphenazine NAA	>0.9999<br><0.0001<br><0.0001<br>>0.9999<br>0.0001<br><0.0001<br><0.0001<br>0.3380	ns<br>***<br>***<br>ns<br>***<br>***<br>***<br>ns
Figure 4J		
control vs. doxycycline<br>control vs. 893<br>control vs. importazole<br>893 vs. 893 doxycycline<br>893 vs. importazole<br>importazole vs. importazole doxycycline<br>doxycycline vs. 893 doxycycline<br>doxycycline vs. importazole doxycycline<br>893 doxycycline vs. importazole doxycycline	0.0005<br><0.0001<br><0.0001<br>>0.9999<br>0.0003<br><0.0001<br><0.0001<br>0.2743<br><0.0001	**<br>***<br>***<br>ns<br>***<br>***<br>***<br>ns<br>***
Figure 5A – Ppp2r1a		
0 µM vs. 10 µM 893<br>0 µM vs. 15 µM 893<br>0 µM vs. 20 µM 893<br>0 µM vs. 30 µM 893<br>0 µM vs. 20 µM sphingosine<br>0 µM vs. 40 µM sphingosine<br>0 µM vs. 60 µM sphingosine<br>0 µM vs. 80 µM sphingosine<br>0 µM vs. 100 µM sphingosine<br>0 µM vs. 60 µM d18:1/6:0 ceramide<br>0 µM vs. 100 µM d18:1/6:0 ceramide	<0.0001<br><0.0001<br><0.0001<br><0.0001<br><0.0001<br><0.0001<br><0.0001<br><0.0001<br><0.0001<br>0.0317<br>0.0321	***<br>***<br>***<br>***<br>***<br>***<br>***<br>***<br>***<br>*<br>*
Figure 5B – Kpnb1		
0 µM vs. 10 µM 893<br>0 µM vs. 15 µM 893<br>0 µM vs. 20 µM 893<br>0 µM vs. 30 µM 893<br>0 µM vs. 10 µM sphingosine<br>0 µM vs. 20 µM sphingosine<br>0 µM vs. 40 µM sphingosine<br>0 µM vs. 60 µM sphingosine<br>0 µM vs. 80 µM sphingosine<br>0 µM vs. 100 µM sphingosine<br>0 µM vs. 20 µM d18:1/2:0 ceramide<br>0 µM vs. 40 µM d18:1/2:0 ceramide<br>0 µM vs. 60 µM d18:1/2:0 ceramide<br>0 µM vs. 80 µM d18:1/2:0 ceramide<br>0 µM vs. 100 µM d18:1/2:0 ceramide<br>0 µM vs. 10 µM d18:1/6:0 ceramide<br>0 µM vs. 20 µM d18:1/6:0 ceramide<br>0 µM vs. 40 µM d18:1/6:0 ceramide<br>0 µM vs. 60 µM d18:1/6:0 ceramide<br>0 µM vs. 80 µM d18:1/6:0 ceramide<br>0 µM vs. 100 µM d18:1/6:0 ceramide	<0.0001<br><0.0001<br><0.0001<br><0.0001<br>0.0016<br><0.0001<br><0.0001<br><0.0001<br><0.0001<br><0.0001<br>0.0032<br><0.0001<br><0.0001<br><0.0001<br>0.0006<br>0.0058<br>0.0001<br>0.0001<br>0.0001<br>0.0001<br>0.0001	***<br>***<br>***<br>***<br>**<br>***<br>***<br>***<br>***<br>***<br>**<br>***<br>***<br>***<br>***<br>**<br>***<br>***<br>***<br>***<br>***
Figure 5E - sphingosine		

SK1 WT 0 vs. 15 min	<0.0001	***
SK1 WT 0 vs. 30 min	<0.0001	***
SK1 WT 0 vs. 60 min	<0.0001	***
SK1 WT 0 vs. 180 min	<0.0001	***
SK1 WT 0 vs. 360 min	0.0357	*
SK1 KO 0 vs. 15 min	<0.0001	***
SK1 KO 0 vs. 30 min	<0.0001	***
SK1 KO 0 vs. 60 min	<0.0001	***
SK1 KO 0 vs. 180 min	<0.0001	***
SK1 KO 0 vs. 360 min	<0.0001	***
SK1 WT 15 min vs. SK1 KO 15 min	0.0114	**
SK1 WT 30 min vs. SK1 KO 30 min	0.0125	**
SK1 WT 60 min vs. SK1 KO 60 min	<0.0001	***
SK1 WT 180 min vs. SK1 KO 180 min	0.0001	***
SK1 WT 360 min vs. SK1 KO 360 min	<0.0001	***
Figure 5F – S1P		
SK1 WT 0 vs. 15 min	0.0053	**
SK1 WT 0 vs. 30 min	<0.0001	***
SK1 WT 0 vs. 60 min	<0.0001	***
SK1 WT 0 vs. 180 min	<0.0001	***
SK1 WT 0 vs. 360 min	<0.0001	***
SK1 KO 0 vs. 15 min	>0.9999	ns
SK1 KO 0 vs. 30 min	>0.9999	ns
SK1 KO 0 vs. 60 min	0.0026	**
SK1 KO 0 vs. 180 min	<0.0001	***
SK1 KO 0 vs. 360 min	<0.0001	***
SK1 WT 15 min vs. SK1 KO 15 min	0.0023	**
SK1 WT 30 min vs. SK1 KO 30 min	0.0004	***
SK1 WT 60 min vs. SK1 KO 60 min	<0.0001	***
SK1 WT 180 min vs. SK1 KO 180 min	<0.0001	***
SK1 WT 360 min vs. SK1 KO 360 min	0.0002	***
Figure 5H		
SK1 WT control vs. sphingosine	0.1793	ns
SK1 WT control vs. SK1 KO control	>0.9999	ns
SK1 KO control vs. sphingosine	<0.0001	***
SK1 WT sphingosine vs. SK1 KO sphingosine	<0.0001	***
Figure 5J		
control vs epoxomicin	>0.9999	ns
control vs. 893	<0.0001	***
control vs. sphingosine	<0.0001	***
control vs. perphenazine	<0.0001	***
893 vs. 893 epoxomicin	0.0078	**
sphingosine vs. sphingosine epoxomicin	<0.0001	***
perphenazine vs. perphenazine epoxomicin	<0.0001	***
epoxomicin vs. 893 epoxomicin	<0.0001	***
epoxomicin vs. sphingosine epoxomicin	<0.0001	***
epoxomicin vs. perphenazine epoxomicin	0.0132	*
893 epoxomicin vs. perphenazine epoxomicin	<0.0001	***
sphingosine epoxomicin vs. perphenazine epoxomicin	<0.0001	***
Figure 5L - SK1 WT		
control vs. TNFa	<0.0001	***
control vs. TNFa 893	0.1060	ns
control vs. TNFa sphingosine	<0.0001	***
TNFa vs. TNFa 893	<0.0001	***
TNFa vs. TNFa sphingosine	<0.0001	***
TNFa 893 vs. TNFa sphingosine	<0.0001	***
Figure 5M – SK1 KO		
control vs. TNFa	<0.0001	***
control vs. TNFa 893	<0.0001	***

control vs. TNFa sphingosine	<0.0001	***
TNFa vs. TNFa 893	<0.0001	***
TNFa vs. TNFa sphingosine	<0.0001	***
TNFa 893 vs. TNFa sphingosine	0.0106	*
Figure 6C - sphingosine		
0 vs. 1 h	0.0027	**
0 vs. 6 h	0.0175	*
Figure 6D – S1P		
0 vs. 1 h	0.1215	ns
0 vs. 6 h	0.0015	**
Figure 6F		
control vs. TNFa	<0.0001	***
TNFa vs. TNFa 893	<0.0001	***
TNFs vs. TNFa PF543	<0.0001	***
Figure 6G		
0 vs. 6 h	0.9447	ns
0 vs. 12 h	0.0020	**
0 vs. 24 h	0.0002	***
0 vs. 36 h	<0.0001	***
Figure 6I - JUN		
control vs. doxycycline	<0.0001	***
control vs. 893	<0.0001	***
Figure 6J - YAP		
control vs. doxycycline	<0.0001	***
control vs. 893	<0.0001	***
Appendix Figure S3A – Ppp2r1a		
0 µM vs. 10 µM 893	0.0006	**
0 µM vs. 20 µM 893	<0.0001	***
0 µM vs. 40 µM 893	<0.0001	***
0 µM vs. 10 µM FTY720	0.4750	ns
0 µM vs. 20 µM FTY720	0.0113	*
0 µM vs. 40 µM FTY720	0.0015	**
Appendix Figure S3B – Kpnb1		
0 µM vs. 10 µM 893	0.0169	*
0 µM vs. 20 µM 893	<0.0001	***
0 µM vs. 40 µM 893	<0.0001	***
0 µM vs. 10 µM FTY720	0.1894	ns
0 µM vs. 20 µM FTY720	0.0007	***
0 µM vs. 40 µM FTY720	<0.0001	***
Appendix Figure S3E		
**Ppp2r1a**		
0 µM vs. 2.5 µM 893	0.9357	ns
0 µM vs. 5 µM 893	0.1496	ns
0 µM vs. 10 µM 893	<0.0001	***
0 µM vs. 15 µM 893	<0.0001	***
0 µM vs. 20 µM 893	<0.0001	***
0 µM vs. 5 µM TX100	0.8269	ns
0 µM vs. 10 µM TX100	>0.9999	ns
0 µM vs. 20 µM TX100	0.8269	ns
**Kpnb1**		
0 µM vs. 2.5 µM 893	>0.9999	ns
0 µM vs. 5 µM 893	0.0189	*
0 µM vs. 10 µM 893	<0.0001	***
0 µM vs. 15 µM 893	<0.0001	***
0 µM vs. 20 µM 893	<0.0001	***
0 µM vs. 5 µM TX100	>0.9999	ns
0 µM vs. 10 µM TX100	>0.9999	ns
0 µM vs. 20 µM TX100	>0.9999	ns

**citrate synthase**		
0 µM vs. 5 µM 893	>0.9999	ns
0 µM vs. 10 µM 893	>0.9999	ns
0 µM vs. 20 µM 893	0.3207	ns
Appendix Figure S3J		
**Ppp2r1a**		
0 µM vs. 0.5 M urea	0.9997	ns
0 µM vs. 1 M urea	0.1607	ns
0 µM vs. 1.25 M urea	0.0022	**
0 µM vs. 1.5 M urea	<0.0001	***
0 µM vs. 1.75 M urea	<0.0001	***
0 µM vs. 2.0 M urea	<0.0001	***
**Kpnb1**		
0 µM vs. 0.5 M urea	0.5685	ns
0 µM vs. 1 M urea	0.0075	**
0 µM vs. 1.25 M urea	<0.0001	***
0 µM vs. 1.5 M urea	<0.0001	***
0 µM vs. 1.75 M urea	<0.0001	***
0 µM vs. 2.0 M urea	<0.0001	***
Appendix Figure S3K – Ppp2r1a		
0 µM vs. 2.5 µM 893	0.9636	ns
0 µM vs. 5 µM 893	0.1800	ns
0 µM vs. 10 µM 893	<0.0001	***
0 µM vs. 15 µM 893	<0.0001	***
0 µM vs. 20 µM 893	<0.0001	***
0 µM vs. 30 µM 893	<0.0001	***
0 µM vs. 5 µM perphenazine	0.0317	*
0 µM vs. 10 µM perphenazine	0.0771	ns
0 µM vs. 20 µM perphenazine	0.5636	ns
0 µM vs. 40 µM perphenazine	0.991	ns
0 µM vs. 60 µM perphenazine	0.9524	ns
0 µM vs. 80 µM perphenazine	0.7513	ns
0 µM vs. 100 µM perphenazine	0.0442	*
0 µM vs. 5 µM DT-061	0.9950	ns
0 µM vs. 10 µM DT-061	0.9950	ns
0 µM vs. 20 µM DT-061	>0.9999	ns
0 µM vs. 40 µM DT-061	>0.9999	ns
0 µM vs. 60 µM DT-061	>0.9999	ns
0 µM vs. 80 µM DT-061	>0.9999	ns
0 µM vs. 100 µM DT-061	0.1250	ns
Appendix Figure S3L – Kpnb1		
0 µM vs. 2.5 µM 893	>0.9999	ns
0 µM vs. 5 µM 893	0.3822	ns
0 µM vs. 10 µM 893	0.0007	***
0 µM vs. 15 µM 893	<0.0001	***
0 µM vs. 20 µM 893	<0.0001	***
0 µM vs. 30 µM 893	<0.0001	***
0 µM vs. 40 µM 893	<0.0001	***
0 µM vs. 5 µM perphenazine	0.9842	ns
0 µM vs. 10 µM perphenazine	0.9842	ns
0 µM vs. 20 µM perphenazine	0.9842	ns
0 µM vs. 30 µM perphenazine	>0.9999	ns
0 µM vs. 40 µM perphenazine	>0.9999	ns
0 µM vs. 60 µM perphenazine	0.6529	ns
0 µM vs. 80 µM perphenazine	0.2357	ns
0 µM vs. 100 µM perphenazine	0.0098	**
0 µM vs. 5 µM DT-061	>0.9999	ns
0 µM vs. 10 µM DT-061	>0.9999	ns
0 µM vs. 20 µM DT-061	>0.9999	ns

0 μM vs. 30 μM DT-061	>0.9999	ns
0 μM vs. 40 μM DT-061	>0.9999	ns
0 μM vs. 60 μM DT-061	>0.9999	ns
0 μM vs. 80 μM DT-061	>0.9999	ns
0 μM vs. 100 μM DT-061	0.4412	ns
Appendix Figure S3M – Ppp2r1a		
5 μM 893 vs. 5 μM 893-ketone	0.4266	ns
40 μM 893 vs. 40 μM 893-ketone	0.3355	ns
100 μM 893 vs. 100 μM 893-ketone	0.0063	**
Appendix Figure S3N – Kpnb1		
5 μM 893 vs. 5 μM 893-ketone	0.3979	ns
40 μM 893 vs. 40 μM 893-ketone	0.0128	*
100 μM 893 vs. 100 μM 893-ketone	0.0001	***
Appendix Figure S7C		
YAP control vs. 893	0.3251	ns
MYC control vs. 893	0.9669	ns
JUN control vs. 893	0.0112	**
hnRNPA1 control vs. 893	0.5621	ns
Appendix Figure S7D - YAP		
DMSO vs. 5 μM 893-alk	<0.0001	***
DMSO vs. 5 μM 893-ketone-alk	<0.0001	***
DMSO vs. 50 μM 893-ketone-alk	<0.0001	***
DMSO vs. 5 μM 893-lactam-alk	>0.9999	ns
DMSO vs. 50 μM 893-lactam-alk	>0.9999	ns
DMSO vs. 5 μM 893-NAc-alk	>0.9999	ns
DMSO vs. 50 μM 893-NAc-alk	0.0044	**
DMSO vs. 5 μM LS-200-alk	<0.0001	***
DMSO vs. 5 μM LS-200-ketone-alk	0.0397	*
DMSO vs. 50 μM LS-200-ketone-alk	<0.0001	***
DMSO vs. 5 μM FTY720-alk	<0.0001	***
DMSO vs. 5 μM FTY720-NAc-alk	0.0004	***
DMSO vs. 50 μM FTY720-NAc-alk	<0.0001	***
DMSO vs. 5 μM PHS-alk	<0.0001	***
DMSO vs. 5 μM PHS-NAc-alk	>0.9999	ns
DMSO vs. 50 μM PHS-NAc-alk	0.0006	***
5 μM 893-alk vs. 5 μM 893-ketone-alk	<0.0001	***
5 μM 893-alk vs. 50 μM 893-ketone-alk	<0.0001	***
5 μM 893-alk vs. 5 μM 893-lactam-alk	<0.0001	***
5 μM 893-alk vs. 50 μM 893-lactam-alk	<0.0001	***
5 μM 893-alk vs. 5 μM 893-NAc-alk	<0.0001	***
5 μM 893-alk vs. 50 μM 893-NAc-alk	<0.0001	***
5 μM LS-200-alk vs. 5 μM LS-200-ketone-alk	<0.0001	***
5 μM LS-200-alk vs. 50 μM LS-200-ketone-alk	<0.0001	***
5 μM FTY720-alk vs. 5 μM FTY720-NAc-alk	<0.0001	***
5 μM FTY720-alk vs. 50 μM FTY720-NAc-alk	<0.0001	***
5 μM PHS-alk vs. 5 μM PHS-NAc-alk	<0.0001	***
5 μM PHS-alk vs. 50 μM PHS-NAc-alk	<0.0001	***
Appendix Figure S7E - JUN		
DMSO vs. 5 μM 893-alk	<0.0001	***
DMSO vs. 5 μM 893-ketone-alk	>0.9999	ns
DMSO vs. 50 μM 893-ketone-alk	>0.9999	ns
DMSO vs. 5 μM 893-lactam-alk	>0.9999	ns
DMSO vs. 50 μM 893-lactam-alk	>0.9999	ns
DMSO vs. 5 μM 893-NAc-alk	0.1536	ns
DMSO vs. 50 μM 893-NAc-alk	>0.9999	ns
DMSO vs. 5 μM LS-200-alk	<0.0001	***
DMSO vs. 5 μM LS-200-ketone-alk	0.4440	ns
DMSO vs. 50 μM LS-200-ketone-alk	<0.0001	***
DMSO vs. 5 μM FTY720-alk	<0.0001	***

DMSO vs. 5 µM FTY720-NAc-alk	0.2853	ns
DMSO vs. 50 µM FTY720-NAc-alk	0.0058	**
DMSO vs. 5 µM PHS-alk	<0.0001	***
DMSO vs. 5 µM PHS-NAc-alk	0.0099	**
DMSO vs. 50 µM PHS-NAc-alk	0.0004	***
5 µM 893-alk vs. 5 µM 893-ketone-alk	<0.0001	***
5 µM 893-alk vs. 50 µM 893-ketone-alk	<0.0001	***
5 µM 893-alk vs. 5 µM 893-lactam-alk	<0.0001	***
5 µM 893-alk vs. 50 µM 893-lactam-alk	<0.0001	***
5 µM 893-alk vs. 5 µM 893-NAc-alk	<0.0001	***
5 µM 893-alk vs. 50 µM 893-NAc-alk	<0.0001	***
5 µM LS-200-alk vs. 5 µM LS-200-ketone-alk	<0.0001	***
5 µM LS-200-alk vs. 50 µM LS-200-ketone-alk	0.2804	ns
5 µM FTY720-alk vs. 5 µM FTY720-NAc-alk	<0.0001	***
5 µM FTY720-alk vs. 50 µM FTY720-NAc-alk	<0.0001	***
5 µM PHS-alk vs. 5 µM PHS-NAc-alk	<0.0001	***
5 µM PHS-alk vs. 50 µM PHS-NAc-alk	<0.0001	***
Appendix Figure S8C		
Vehicle vs. 893	<0.0001	***
Appendix Figure S8E		
GFP control vs. 893	0.7718	ns
GFP-AR-V7 control vs. 893	<0.0001	***
Appendix Figure S8F		
p21 control vs. 893	0.0489	*
p27 control vs. 893	0.0335	*
GAS1 control vs. 893	0.0051	**
ODC1 control vs. 893	<0.0001	***
PAICS control vs. 893	0.6349	ns
LDHA control vs. 893	0.0005	***
CCND2 control vs. 893	0.0012	***
DNMT1 control vs. 893	0.0853	ns
Appendix Figure S8G		
FKBP5 control vs. 893	0.0022	**
Deptor control vs. 893	0.0241	*
TMPRSS2 control vs. 893	0.0656	ns
KLK3 control vs. 893	0.0208	*
Appendix Figure S9E		
doxycycline vs. doxycycline 893	<0.0001	***
doxycycline vs. doxycycline epoxomicin	0.0020	**
doxycycline vs. doxycycline epoxomicin 893	>0.9999	ns
doxycycline 893 vs. doxycycline epoxomicin	<0.0001	***
doxycycline 893 vs. doxycycline 893 epoxomicin	<0.0001	***
doxycycline epoxomicin vs. doxycycline 893 epoxomicin	0.0653	ns
Appendix Figure S9J		
control vs. epoxomicin	>0.9999	ns
control vs. 893	<0.0001	***
control vs. 893 + epoxomicin	<0.0001	***
control vs. perphenazine	<0.0001	***
control vs. perphenazine + epoxomicin	0.0476	*
control vs. d18:1/2:0 ceramide	<0.0001	***
control vs. d18:1/2:0 ceramide epoxomicin	>0.9999	ns
epoxomicin vs. 893	<0.0001	***
epoxomicin vs. 893 epoxomicin	<0.0001	***
epoxomicin vs. perphenazine	<0.0001	***
epoxomicin vs. perphenazine epoxomicin	>0.9999	ns
epoxomicin vs. d18:1/2:0 ceramide	<0.0001	***
epoxomicin vs. d18:1/2:0 ceramide epoxomicin	>0.9999	ns
893 vs. epoxomicin	>0.9999	ns
893 vs. perphenazine	>0.9999	ns

893 vs. perphenazine epoxomicin	<0.0001	***
893 vs. d18:1/2:0 ceramide	>0.9999	ns
893 vs. d18:1/2:0 ceramide epoxomicin	<0.0001	***
893 epoxomicin vs. perphenazine	>0.9999	ns
893 epoxomicin vs. perphenazine epoxomicin	<0.0001	***
893 epoxomicin vs. d18:1/2:0 ceramide	0.1107	ns
893 epoxomicin vs. d18:1/2:0 ceramide epoxomicin	<0.0001	***
perphenazine vs. perphenazine epoxomicin	<0.0001	***
perphenazine vs. d18:1/2:0 ceramide	>0.9999	ns
perphenazine vs. d18:1/2:0 ceramide epoxomicin	<0.0001	***
perphenazine epoxomicin vs. d18:1/2:0 ceramide	<0.0001	***
perphenazine epoxomicin vs. d18:1/2:0 ceramide epoxomicin	0.8977	ns
d18:1/2:0 ceramide vs. d18:1/2:0 ceramide epoxomicin	<0.0001	***
Appendix Figure S10C		
**Ppp2r1a**		
0 µM vs. 10 µM 3-keto-sphinganine	<0.0001	***
0 µM vs. 20 µM 3-keto-sphinganine	<0.0001	***
0 µM vs. 40 µM 3-keto-sphinganine	<0.0001	***
0 µM vs. 10 µM d12:1 4E sphingosine	0.9921	ns
0 µM vs. 20 µM d12:1 4E sphingosine	0.9720	ns
0 µM vs. 40 µM d12:1 4E sphingosine	0.7259	ns
0 µM vs. 10 µM d20:1 4E sphingosine	0.0016	**
0 µM vs. 20 µM d20:1 4E sphingosine	0.0002	***
0 µM vs. 40 µM d20:1 4E sphingosine	<0.0001	***
0 µM vs. 10 µM d18:1 4E sphingosine	0.0068	**
0 µM vs. 20 µM d18:1 4E sphingosine	<0.0001	***
0 µM vs. 40 µM d18:1 4E sphingosine	<0.0001	***
0 µM vs. 10 µM d18:1 14Z sphingosine	0.0210	*
0 µM vs. 20 µM d18:1 14Z sphingosine	<0.0001	***
0 µM vs. 40 µM d18:1 14Z sphingosine	<0.0001	***
10 µM d18:1 4E sphingosine vs. 10 µM d18:1 14Z sphingosine	0.2222	ns
20 µM d18:1 4E sphingosine vs. 20 µM d18:1 14Z sphingosine	0.0008	***
40 µM d18:1 4E sphingosine vs. 40 µM d18:1 14Z sphingosine	0.0009	***
10 µM d18:1 4E sphingosine vs. 10 µM d12:1 14E sphingosine	0.0007	***
20 µM d18:1 4E sphingosine vs. 20 µM d12:1 14E sphingosine	0.0002	***
40 µM d18:1 4E sphingosine vs. 40 µM d12:1 14E sphingosine	<0.0001	***
10 µM d18:1 4E sphingosine vs. 10 µM d20:1 14E sphingosine	0.9659	ns
20 µM d18:1 4E sphingosine vs. 20 µM d20:1 14E sphingosine	0.0014	***
40 µM d18:1 4E sphingosine vs. 40 µM d20:1 14E sphingosine	<0.0001	***
10 µM d18:1 4E sphingosine vs. 10 µM 3-keto-sphinganine	0.0192	*
20 µM d18:1 4E sphingosine vs. 20 µM 3-keto-sphinganine	0.0043	**
40 µM d18:1 4E sphingosine vs. 40 µM 3-keto-sphinganine	0.0001	***
**Kpnb1**		
0 µM vs. 10 µM 3-keto-sphinganine	<0.0001	***
0 µM vs. 20 µM 3-keto-sphinganine	<0.0001	***
0 µM vs. 40 µM 3-keto-sphinganine	<0.0001	***
0 µM vs. 10 µM d12:1 4E sphingosine	0.4318	ns
0 µM vs. 20 µM d12:1 4E sphingosine	0.2349	ns
0 µM vs. 40 µM d12:1 4E sphingosine	0.1597	ns
0 µM vs. 10 µM d20:1 4E sphingosine	0.0033	**
0 µM vs. 20 µM d20:1 4E sphingosine	0.0009	***
0 µM vs. 40 µM d20:1 4E sphingosine	0.0003	***
0 µM vs. 10 µM d18:1 4E sphingosine	0.5999	ns
0 µM vs. 20 µM d18:1 4E sphingosine	0.0006	***
0 µM vs. 40 µM d18:1 4E sphingosine	<0.0001	***
0 µM vs. 10 µM d18:1 14Z sphingosine	0.5303	ns
0 µM vs. 20 µM d18:1 14Z sphingosine	0.0002	***
0 µM vs. 40 µM d18:1 14Z sphingosine	<0.0001	***
10 µM d18:1 4E sphingosine vs. 10 µM d18:1 14Z sphingosine	0.2588	ns
20 µM d18:1 4E sphingosine vs. 20 µM d18:1 14Z sphingosine	0.0016	**

40 µM d18:1 4E sphingosine vs. 40 µM d18:1 14Z sphingosine	0.0605	ns
10 µM d18:1 4E sphingosine vs. 10 µM d12:1 14E sphingosine	0.0327	*
20 µM d18:1 4E sphingosine vs. 20 µM d12:1 14E sphingosine	<0.0001	***
40 µM d18:1 4E sphingosine vs. 40 µM d12:1 14E sphingosine	0.0018	**
10 µM d18:1 4E sphingosine vs. 10 µM d20:1 14E sphingosine	0.1462	ns
20 µM d18:1 4E sphingosine vs. 20 µM d20:1 14E sphingosine	0.0012	***
40 µM d18:1 4E sphingosine vs. 40 µM d20:1 14E sphingosine	0.0050	**
10 µM d18:1 4E sphingosine vs. 10 µM 3-keto-sphinganine	0.0061	**
20 µM d18:1 4E sphingosine vs. 20 µM 3-keto-sphinganine	0.0031	**
40 µM d18:1 4E sphingosine vs. 40 µM 3-keto-sphinganine	0.0081	**
