# Supplementary material for: Sphingosine simultaneously inhibits nuclear import and activates PP2A by binding importins and PPP2R1A
Source: EMBO J. 2025 Jun 30;44(16):4473–98. doi: 10.1038/s44318-025-00490-5 (PMC12361511; doi:10.1038/s44318-025-00490-5)
Supplement: Supplementary file 2 — Table EV1 [file 44318_2025_490_MOESM2_ESM.docx]

**Table EV1:** Ribosomal proteins that are lost from the

nucleus upon SH-BC-893 treatment or isolated with 893-diazirine

| Ribosomal protein | ↓nuclear proteome | 893-PAL | Ribosomal protein | ↓nuclear proteome | 893-PAL |
| --- | --- | --- | --- | --- | --- |
| Nmd3 | X |  | **Rplp0** | X | X |
| Rpl3 | X | X | **Rplp1** | X |  |
| Rpl4 | X | X | **Rplp2** | X |  |
| Rpl5 | X | X | **Rps2** | X | X |
| Rpl6 | X | X | **Rps3** | X | X |
| Rpl7 | X | X | **Rps3a** | X | X |
| Rpl7a | X | X | **Rps4x** | X | X |
| Rpl8 | X | X | **Rps5** | X |  |
| Rpl9 | X | X | **Rps6** | X | X |
| Rpl10 | X | X | **Rps7** | X |  |
| Rpl10a | X |  | **Rps8** | X | X |
| Rpl11 | X |  | **Rps9** | X | X |
| Rpl12 | X |  | **Rps10** | X |  |
| Rpl13 | X | X | **Rps11** | X | X |
| Rpl13a | X | X | **Rps12** | X |  |
| Rpl14 | X |  | **Rps13** | X |  |
| Rpl15 | X | X | **Rps14** | X |  |
| Rpl16 | X |  | **Rps15** | X |  |
| Rpl17 | X |  | **Rps15a** | X |  |
| Rpl18 | X | X | **Rps16** |  | X |
| Rpl18a | X | X | **Rps17** | X | X |
| Rpl19 | X | X | **Rps18** | X |  |
| Rpl21 | X | X | **Rps19** | X |  |
| Rpl22 | X |  | **Rps20** | X |  |
| Rpl22l1 | X |  | **Rps21** | X |  |
| Rpl23a | X |  | **Rps23** | X |  |
| Rpl24 | X | X | **Rps24** | X |  |
| Rpl26 | X | X | **Rps25** | X |  |
| Rpl27a | X |  | **Rps26** | X |  |
| Rpl28 | X | X | **Rps27** | X |  |
| Rpl29 | X |  | **Rps27l** | X |  |
| Rpl30 | X |  | **Rps29** | X |  |
| Rpl31 | X |  | **Rpsa** | X | X |
| Rpl32 | X | X | **Rrbp1** | X |  |
| Rpl34 | X |  | **Rrp8** | X |  |
| Rpl35 | X |  |  |  |  |
| Rpl35a | X | X |  |  |  |
| Rpl36 | X |  |  |  |  |
| Rpl36a | X |  |  |  |  |
| Rpl37a | X |  |  |  |  |
