# Supplementary material for: Sphingosine simultaneously inhibits nuclear import and activates PP2A by binding importins and PPP2R1A
Source: EMBO J. 2025 Jun 30;44(16):4473–98. doi: 10.1038/s44318-025-00490-5 (PMC12361511; doi:10.1038/s44318-025-00490-5)
Supplement: Supplementary file 3 — Table EV2 [file 44318_2025_490_MOESM3_ESM.docx]

**Table EV2:** Metabolic enzymes that are lost from the nucleus upon treatment with SH-BC-893 or that are pulled down by 893-diazirine

|  | name | function | ↓nuclear proteome | 893-PAL |
| --- | --- | --- | --- | --- |
| Hk1 | hexokinase | glycolysis |  | X |
| Gpi | Glucose-6-phosphate isomerase | glycolysis | X | X |
| Pfkl | ATP-dependent 6-phosphofructokinase | glycolysis | X | X |
| Pfkp | ATP-dependent 6-phosphofructokinase | glycolysis |  | X |
| Aldoa | Fructose-bisphosphate aldolase A | glycolysis | X | X |
| Gapdh | glyceraldehyde-3-phosphate dehydrogenase | glycolysis | X | X |
| Pgk1 | Phosphoglycerate kinase 1 | glycolysis | X | X |
| Pgam1 | phosphoglycerate mutase 1 | glycolysis | X | X |
| Pgm1 | Phosphoglucomutase-1 | glycolysis | X |  |
| Eno1 | Alpha enolase | glycolysis | X | X |
| Pkm | pyruvate kinase | glycolysis | X | X |
| Ldha | Lactate dehydrogenase A | glycolysis | X | X |
| Pc | pyruvate carboxylase | Links glycolysis and TCA cycle |  | X |
| Aco2 | aconitate hydratase 2 | TCA cycle |  | X |
| Idh2 | isocitrate dehydrogenase 3 | TCA cycle | X |  |
| Fh | Fumarate hydratase | TCA cycle | X |  |
| Mdh2 | malate dehydrogenase 2 | TCA cycle | X | X |
| Sdha | Succinate dehydrogenase | TCA cycle |  | X |
| Idh3a | isocitrate dehydrogenase 3 | TCA cycle | X | X |
| Tkt | transketolase | PPP | X | X |
| Taldo1 | transaldolase | PPP |  | X |
| Pgd | 6-phosphogluconate dehydrogenase | PPP | X | X |
| G6pdx | glucose-6-phosphate 1-dehydrogenase | PPP | X | X |
| Acly | ATP citrate lyase | TCA → FAS |  | X |
| Fasn | Fatty acid synthase | FA synthesis | X | X |
| Acaca | Acetyl-CoA carboxylase 1 | FA synthesis |  | X |
| Aldh18a1 | Delta-1-pyrroline-5-carboxylate synthase | Proline, ornithine, arginine synthesis |  | X |
| Mthfd1and Mthfd1l | Methylenetetrahydrofolate dehydrogenase, C-1-tetrahydrofolate synthase | One carbon metabolism | X | X |
| Mtap | S-methyl-5-thioadenosine phosphorylase | Adenine and methionine salvage | X |  |
| Phgdh | D-3-phosphoglycerate dehydrogenase | Serine synthesis | X | X |
| Psat1 | phosphoserine aminotransferase | Serine synthesis | X | X |
| Asns | asparagine synthetase | Asparagine synthesis |  | X |
| Got2 | aspartate aminotransferase | Amino acid metabolism, urea cycle, TCA cycle | X | X |
| Aldh3a1 | Aldehyde dehydrogenase | Redox stress | X |  |
| Aldh2 | Aldehyde dehydrogenase | Redox stress |  | X |
